# Hepatitis C co-infection is associated with an increased risk of incident chronic kidney disease in HIV-infected patients initiating combination antiretroviral therapy

**DOI:** 10.1186/s12879-017-2350-8

**Published:** 2017-04-04

**Authors:** Carmine Rossi, Janet Raboud, Sharon Walmsley, Curtis Cooper, Tony Antoniou, Ann N. Burchell, Mark Hull, Jason Chia, Robert S. Hogg, Erica E.M. Moodie, Marina B. Klein, the Canadian Observational Cohort (CANOC) Collaboration, Robert Hogg, Ann N. Burchell, Curtis Cooper, Deborah Kelly, Marina Klein, Mona Loutfy, Nima Machouf, Julio Montaner, Janet Raboud, Chris Tsoukas, Stephen Sanche, Alexander Wong, Tony Antoniou, Ahmed Bayoumi, Mark Hull, Bohdan Nosyk, Angela Cescon, Michelle Cotterchio, Charlie Goldsmith, Silvia Guillemi, P. Richard Harrigan, Marianne Harris, Sean Hosein, Sharon Johnston, Claire Kendall, Clare Liddy, Viviane Lima, David Marsh, David Moore, Alexis Palmer, Sophie Patterson, Peter Phillips, Anita Rachlis, Sean B. Rourke, Hasina Samji, Marek Smieja, Benoit Trottier, Mark Wainberg, Sharon Walmsley, Chris Archibald, Ken Clement, Fred Crouzat, Monique Doolittle-Romas, Laurie Edmiston, Sandra Gardner, Brian Huskins, Jerry Lawless, Douglas Lee, Renee Masching, Stephen Tattle, Alireza Zahirieh, Claire Allen, Stryker Calvez, Jason Chia, Daniel Corsi, Louise Gilbert, Nada Gataric, Alia Leslie, Lucia Light, Costas Pexos, Susan Shurgold, Leah Szadkowski, Chrissi Galanakis, Ina Sandler, Benita Yip, Jaime Younger, Julia Zhu

**Affiliations:** 1 Research Institute of the McGill University Health Centre, Montréal, Canada; 2 Dalla Lana School of Public Health, University of Toronto, Toronto, Canada; 3 Toronto General Hospital Research Institute, University Health Network, Toronto, Canada; 4 The Ottawa Hospital, General Campus, Ottawa, Canada; 5 St. Michael’s Hospital, University of Toronto, Toronto, Canada; 6 BC Centre for Excellence in HIV/AIDS, Vancouver, Canada; 7 Faculty of Medicine, University of British Columbia, Vancouver, Canada; 8 Faculty of Health Sciences, Simon Fraser University, Vancouver, Canada; 9 Department of Epidemiology, Biostatistics and Occupational Health, McGill University, Montréal, Canada; 10 Division of Infectious Diseases and Chronic Viral Illness Service, McGill University Health Centre, 1001 Decarie Boulevard, D02.4110, Montréal, H4A 3J1 Canada

**Keywords:** Antiretroviral therapy, Chronic kidney disease, Co-infection, Glomerular filtration, Hepatitis C, HIV

## Abstract

**Background:**

Combination antiretroviral therapy (cART) has reduced mortality from AIDS-related illnesses and chronic comorbidities have become prevalent among HIV-infected patients. We examined the association between hepatitis C virus (HCV) co-infection and chronic kidney disease (CKD) among patients initiating modern antiretroviral therapy.

**Methods:**

Data were obtained from the Canadian HIV Observational Cohort for individuals initiating cART from 2000 to 2012. Incident CKD was defined as two consecutive serum creatinine-based estimated glomerular filtration (eGFR) measurements <60 mL/min/1.73m^2^ obtained ≥3 months apart. CKD incidence rates after cART initiation were compared between HCV co-infected and HIV mono-infected patients. Hazard ratios (HRs) and 95% confidence intervals (CIs) were estimated using multivariable Cox regression.

**Results:**

We included 2595 HIV-infected patients with eGFR >60 mL/min/1.73m^2^ at cART initiation, of which 19% were HCV co-infected. One hundred and fifty patients developed CKD during 10,903 person-years of follow-up (PYFU). The CKD incidence rate was higher among co-infected than HIV mono-infected patients (26.0 per 1000 PYFU vs. 10.7 per 1000 PYFU). After adjusting for demographics, virologic parameters and traditional CKD risk factors, HCV co-infection was associated with a significantly shorter time to incident CKD (HR 1.97; 95% CI: 1.33, 2.90). Additional factors associated with incident CKD were female sex, increasing age after 40 years, lower baseline eGFR below 100 mL/min/1.73m^2^, increasing HIV viral load and cumulative exposure to tenofovir and lopinavir.

**Conclusions:**

HCV co-infection was associated with an increased risk of incident CKD among HIV-infected patients initiating cART. HCV-HIV co-infected patients should be monitored for kidney disease and may benefit from available HCV treatments.

**Electronic supplementary material:**

The online version of this article (doi:10.1186/s12879-017-2350-8) contains supplementary material, which is available to authorized users.

## Background

Combination antiretroviral therapy (cART) has been associated with both a substantial reduction in AIDS and greater life expectancies in both low and high-income HIV settings [1–3]. With increasing life expectancies, morbidity and mortality from non-AIDS and ageing-related co-morbidities account for a larger proportion of the HIV disease burden [4]. Co-infection with chronic hepatitis C virus (HCV) is common among HIV-infected patients and is associated with many of the extrahepatic, non-AIDS and ageing-related co-morbidities observed in this population [5]. These co-morbidities include cardiovascular, metabolic, renal, and neurological illnesses, which have been attributed to chronic HCV infection in both the general and HIV-infected populations [6–8]. Indeed, the diagnoses of these extrahepatic illnesses have increased among hospitalized patients with HCV and account for a greater proportion of health service utilization among co-infected individuals [9, 10].

Chronic kidney disease (CKD) is an important comorbidity in HIV-infected persons [11]. If left unmanaged, HIV-infected patients with CKD are at greater risk of serious cardiovascular complications and premature mortality [12–14]. The etiology of CKD in HIV-infected patients is complex and has been related to both traditional, non-viral kidney impairment risk factors, such as hypertension, diabetes, dyslipidemia, and the use of nonsteroidal anti-inflammatory drugs (NSAIDs), whose prevalence have all increased in the modern cART era, as well as emerging HIV-related risk factors, such as long-term use of potentially nephrotoxic antiviral agents, incomplete immune recovery, and ongoing substance abuse [15–17]. Chronic HCV has been both directly and indirectly implicated in the development of CKD. Directly, HCV has been associated with the development of glomerulonephritis, via increased autoantibody IgM production with rheumatoid factor activity, leading to mixed cryoglobulinemia and deposition into glomerular capillary and tubules [18, 19]. Indirectly, HCV has been associated with an increased risk of insulin resistance and atherosclerosis, which are important CKD risk factors [20].

Given the complex interplay between viral and non-viral risk factors, the impact that chronic HCV may have on CKD in the modern cART era is unclear. A meta-analysis of 27 studies in HIV-infected individuals enrolled between 1989 and 2006 showed that HCV infection was associated with an increased risk of CKD. However, there was both statistical heterogeneity and variation in measured outcomes (i.e. CKD measured by proteinuria, hospitalization, incidence of HIV-associated nephropathy (HIVAN), or increased creatinine concentration), as well as no adjustment for confounding [21]. Studies included a combination of treatment-naïve and experienced patients, including treatment with indinavir, and therefore may no longer be representative of the HIV-infected population currently in clinical care. The objective of this study was to determine if HCV co-infection is associated with an increased risk of incident CKD in a cohort of HIV-infected Canadians initiating modern antiretroviral therapy.

## Methods

### Study population

Data were analyzed from the Canadian Observational Cohort (CANOC) [22]. This retrospective HIV cohort study is a collaboration of eight separate population and clinic-based cohorts from Canada’s largest provinces. Briefly, treatment-naïve HIV-infected patients who have initiated cART since January 1st, 2000, are eligible for inclusion. The cohort is broadly representative of the HIV population accessing treatment and comprises approximately half of all individuals who have initiated cART in Canada since 2000 [22]. Every second year, each participating cohort electronically submits a pre-defined series of demographic, clinical, and HIV treatment variables for their patients to the Data Coordinating Site at the British Columbia Centre for Excellence in HIV/AIDS Research. As of December 31st, 2012, 8980 cART initiators have been included in CANOC. All participating cohorts have received approval from their institutional ethics boards to contribute anonymous patient data to CANOC.

### Study design and inclusion criteria

A longitudinal cohort design was employed to examine the association between HCV co-infection and incident CKD. We defined baseline as the date of cART initiation and we included all individuals who (1) had at least one serum creatinine (SCr) measurement within 30 days prior to starting cART or up to 7 days after, (2) had at least two follow-up SCr measurements, at least 90 days apart, and (3) did not have a baseline estimated glomerular filtration rate (eGFR) measurement <60 mL/min/1.73 m^2^ (see below).

### Chronic kidney disease

We calculated eGFR using the 2009 SCr-based CKD-EPI equation [23]. This equation has been validated in HIV-infected populations [24]. Confirmed incident CKD was defined as two consecutive eGFR measurements <60 mL/min/1.73 m^2^, obtained at least 90 days apart. The CKD event date was on the second of the two required eGFR measurements.

### Study covariates

HCV exposure was ascertained by a positive HCV antibody test, qualitative or quantitative HCV RNA analysis, or physician reporting. HCV exposure was considered time-fixed irrespective of when the diagnosis or testing results were reported, as HCV infection precedes cART initiation in the vast majority of HIV-infected Canadians [25]. African/Caribbean ethnicity and injection drug use (IDU) as an HIV risk factor were obtained by patient self-report at baseline from patients in the Ontario HIV Treatment Network Cohort Study, from enrolment forms into the Drug Treatment Program in British Columbia, or from review of medical charts for other cohorts. Diabetes was defined by either clinical diagnoses or random serum glucose ≥11.1 mmol/L. Hypertension was also defined by either clinical diagnoses or systolic blood pressure ≥ 140 mmHg or diastolic blood pressure ≥ 90 mmHg [26]. Liver fibrosis was measured using the aspartate aminotransferase-to-platelet ratio index (APRI) and values ≥1.5 were indicative of moderate fibrosis [27]. AIDS-defining events were defined based on the 1993 Centers for Disease Control and Prevention classification [28].

### Statistical analyses

Eligible patients were followed from baseline (i.e. cART initiation) until the earliest of (1) incident CKD, (2) death, (3) loss-to-follow-up, or (4) study end date of December 31st, 2012. Person-time for individuals without CKD was censored at the date of the last eGFR measurement or date of death. Cumulative incidence curves were created to explore the association between HCV and CKD-free survival [29]. Univariable and multivariable Cox proportional hazards (PH) models were fit to examine the association between HCV co-infection and CKD. The multivariable model was adjusted for baseline age, sex, African/Caribbean ethnicity, eGFR, and year of cART initiation and time-updated measurements of CD4^+^ cell count, HIV viral load, liver fibrosis, and tenofovir, atazanavir, and lopinavir use. A second model additionally adjusted for time-updated diabetes and hypertension. Covariates were selected a priori based on their plausibility as confounders. As there was evidence of non-linearity between age and baseline eGFR with CKD, we modeled these covariates with linear splines with a single age knot at 40 years and a single baseline eGFR knot at 100 mL/min/1.73 m^2^ [30]. Chronic comorbid conditions of liver fibrosis, diabetes and hypertension were modeled as cumulative binary exposures and the antiretroviral agents were modeled as cumulative use. We explored interaction between HCV co-infection and IDU as an HIV risk factor, as previous research has shown poorer clinical outcomes for this group [31]. Missing covariate data for HCV co-infection, African/Caribbean ethnicity, liver fibrosis, diabetes and hypertension was handled using fully conditional specification multiple imputation (MI) [32]. The imputation model included all covariates in the multivariable model, an indicator for CKD, and a measure of the cumulative baseline hazard using the Nelson-Aalen estimator [33]. We created 10 imputed data sets and combined regression results using Rubin’s rules [32].

We also performed several sensitivity analyses. First, given there were a large proportion of patients without a baseline eGFR that were excluded from the analysis, we used inverse-probability weighting to account for the fact that certain patients were under-represented in our analytical cohort [34]. Second, because there is a strong relationship between increasing age and declining kidney function, we considered using age as the time scale in the primary Cox PH model. Third, we considered reclassifying exposed co-infected person-time based on the date of first positive HCV serologic or molecular test, rather than assume patients were initially co-infected [35]. Lastly, given the large number of missing data on hypertension, we examined the sensitivity of the missing at random MI assumption [36]. STATA version 13.1 (College Station, TX) and SAS version 9.4 (Cary, NC) were used for the analyses.

## Results

**Fig. 1:**
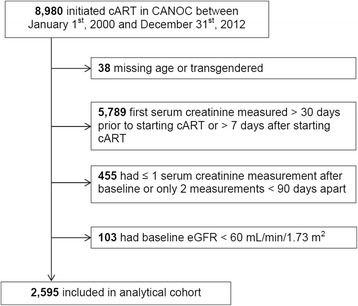
Study inclusion criteria

Of the 8980 patients who initiated cART between January 1st, 2000, and December 31st, 2012, 2595 (29%) were eligible for inclusion into our study (Fig. 1). Most patients were excluded (64%) because they did not have a baseline SCr measurement. Excluded individuals were more likely to be female, white, HCV co-infected, report IDU as an HIV risk factor, and used tenofovir-based initial cART regimen (see Additional file 1: Table S1).

Baseline patient characteristics, overall and stratified by HCV co-infection, are presented in Table 1. Most of the study patients were male (85%) and the median age was 40 years (interquartile range [IQR]: 33, 46). Among the 2595 included patients, 2462 (95%) were screened for HCV. Of these, 484 (20%) were co-infected, with 107 (22%) could be confirmed as having chronic HCV with molecular HCV RNA testing. Compared to HIV mono-infected, co-infected patients were older, less likely to be male or of African/Caribbean ethnicity, more likely to have a history of IDU and have liver fibrosis, less likely to be on a tenofovir-based initial cART regimen and had lower baseline CD4^+^ cell counts.

**Table 1:** Baseline study characteristics, overall and stratified by hepatitis C virus co-infection

	Overall (*n* = 2595)	HCV positive (*n* = 484)	HCV negative (*n* = 1978)	Unknown (*n* = 133)
Median age (IQR), years	40 (33, 46)	41 (35, 47)	39 (33, 46)	41 (36, 49)
Male sex	2195 (85%)	394 (81%)	1690 (85%)	111 (83%)
African/Caribbean ethnicity ^a^	368 (24%)	17 (5%)	344 (29%)	17 (27%)
Median eGFR (IQR), mL/min/1.73 m^2^	105 (92, 116)	103 (91, 114)	105 (92, 117)	106 (96, 115)
Injection drug use as HIV risk factor ^b^	389 (18%)	302 (69%)	83 (5%)	4 (4%)
Median CD4^+^ cell count (IQR), cells/μL	210 (102, 318)	190 (80, 290)	220 (110, 323)	200 (100, 319)
Median HIV viral load (IQR), log_10_ copies/mL	4.9 (4.4, 5.2)	4.9 (4.4, 5.1)	4.9 (4.4, 5.2)	4.9 (4.4, 5.2)
Previous AIDS-defining event ^c^	450 (19%)	99 (22%)	331 (19%)	20 (18%)
Tenofovir use	1410 (54%)	227 (47%)	1111 (56%)	72 (54%)
Atazanavir use	666 (26%)	144 (30%)	494 (25%)	28 (21%)
Lopinavir use	471 (18%)	99 (20%)	347 (18%)	25 (19%)
Median year of cART initiation (IQR)	2007 (2004, 2009)	2006 (2003, 2009)	2008 (2004, 2009)	2008 (2004, 2010)
Liver fibrosis (APRI ≥1.5) ^d^	145 (7%)	69 (17%)	71 (5%)	5 (6%)
Diabetes ^e^	119 (5%)	26 (6%)	89 (5%)	4 (3%)
Hypertension ^f^	74 (12%)	13 (15%)	59 (12%)	2 (8%)
Cohort province				
British Columbia	1103 (43%)	307 (63%)	738 (37%)	58 (44%)
Ontario	838 (32%)	110 (23%)	694 (35%)	34 (26%)
Québec	654 (25%)	67 (14%)	546 (28%)	41 (31%)

*APRI* aspartate aminotransferase to platelet ratio index, *cART* combination antiretroviral therapy, *IQR* interquartile range, *eGFR* estimated glomerular filtration rate, *HCV* hepatitis C virus

^a^ 1045 participants (40%) are missing data on African/Caribbean ethnicity; 138 among HCV Positive, 838 among HCV Negative, and 69 among the Unknown

^b^ 431 participants (17%) are missing data on injection drug use risk factor; 44 among HCV Positive, 344 among HCV Negative, and 43 among the Unknown

^c^ 246 participants (9%) are missing previous AIDS event histories; 31 among HCV Positive, 197 among HCV Negative, and 18 among the Unknown

^d^ 603 participants (23%) are missing data on liver fibrosis; 73 among HCV Positive, 484 among HCV Negative, and 46 among the Unknown

^e^ 142 participants (5%) are missing data on diabetes; 13 among HCV Positive, 111 among HCV Negative, and 18 among the Unknown

^f^ 1973 participants (76%) are missing data on hypertension; 395 among HCV Positive, 1470 among HCV Negative, and 108 among the Unknown

Of the 2595 patients in the study, 150 (6%) developed incident CKD over a total of 10,903 person-years of follow-up (PYFU). The overall crude incidence rate was 13.8 per 1000 PYFU (95% confidence interval (CI): 11.7, 16.1). The median follow-up time was 3.5 years (IQR: 1.7, 6.1). Of the 2445 patients who were censored without developing CKD, 141 died (6%), 576 were lost-to-follow-up (23%), and the remaining 1728 were administratively censored (71%).

**Fig. 2:**
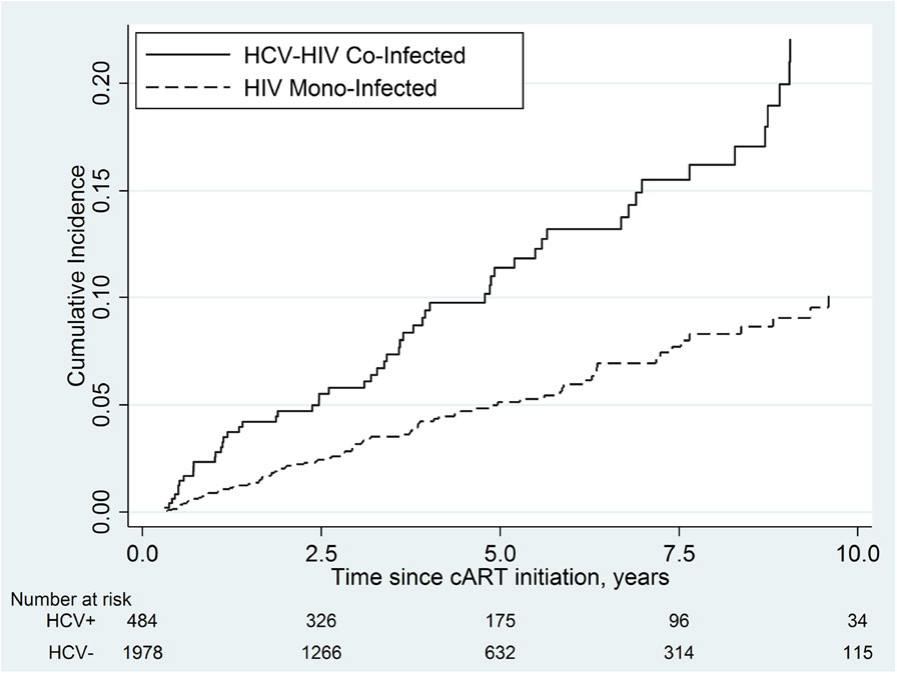
Cumulative incidence of chronic kidney disease by hepatitis C co-infection

Incidence rates for CKD stratified by demographic and clinical characteristics are presented in Table 2. Crude rates were higher in females, older age groups, non-African/Caribbeans, individuals with IDU as an HIV risk factor, and those with a lower baseline eGFR. CKD incidence did not increase with calendar time. The incidence of CKD among co-infected patients was substantially higher than the rate among HIV mono-infected patients; 26.0 per 1000 PYFU (95% CI: 20.0, 33.8) and 10.7 per 1000 PYFU (95% CI: 8.7, 13.2), respectively. The median follow-up time was similar in both groups. The incidence rate of CKD among those with an unknown HCV status was similar to the HIV mono-infected group (11.1 per 1000 PYFU; 95% CI: 4.6, 26.7). Figure 2 depicts the cumulative incidence function for CKD by HCV co-infection status. Among co-infected, the five-year cumulative risk of CKD after initiating cART was 11%, compared to 5% among HIV mono-infected patients.

**Table 2:** Crude incidence rates of chronic kidney disease, Canadian Observational Cohort 2000–2012

Characteristic	Chronic kidney Disease events	Total Person-years	Incidence rate per 1000 Person-years (95% CI)
Overall	150	10,903.4	13.8 (11.7, 16.1)
Sex			
Male	115	9130.4	12.6 (10.5, 15.1)
Female	35	1773.0	19.7 (14.2, 27.5)
Ethnicity			
African/Caribbean	15	1806.6	8.3 (5.0, 13.7)
Non-African/Caribbean	97	5742.7	16.9 (13.8, 20.6)
Unknown	38	3354.0	11.3 (8.2, 15.6)
Hepatitis C Co-Infection			
Yes	56	2156.0	26.0 (20.0, 33.8)
No	89	8297.3	10.7 (8.7, 13.2)
Unknown	5	450.2	11.1 (4.6, 26.7)
HIV Risk Factor			
Injection drug use	45	1709.7	25.1 (18.8, 33.7)
Non-injection drug use	89	7640.4	11.6 (9.5, 14.3)
Unknown	16	1472.3	10.9 (6.7, 17.7)
Age at cART initiation, years			
18–39	43	5342.6	8.0 (6.0, 10.9)
40–49	41	3874.6	10.6 (7.8, 14.4)
50–59	40	1369.5	29.2 (21.4, 39.8)
≥ 60	26	316.7	82.1 (55.9, 120.6)
Baseline eGFR, mL/min/1.73m^2^			
> 110	25	3906.9	6.4 (4.3, 9.5)
> 90 & ≤ 110	41	4405.5	9.3 (6.9, 12.6)
≤ 90	84	2591.0	32.4 (26.2, 40.2)
Year of Follow-up			
2000–2003	14	966.4	14.5 (8.6, 24.5)
2004–2008	58	4324.0	13.4 (10.4, 17.3)
2009–2012	78	5613.0	13.9 (11.1, 17.3)

*cART* combination antiretroviral therapy, *CI* confidence interval, *eGFR* estimated glomerular filtration rate

In univariable analysis, HCV co-infection was significantly associated with CKD (hazard ratio (HR) 2.49; 95% CI: 1.79, 3.48) and remained so after adjustment (HR 1.97; 95% CI: 1.33, 2.90). Female sex (HR 2.16; 95% CI: 1.42, 3.28), increasing age after 40 years (HR 1.51 per 5 year increase; 95% CI: 1.35, 1.67), increasing baseline eGFR up to 100 mL/min/1.73 m^2^ (HR 0.60 per 5 mL/min/1.73 m^2^ increase; 95% CI: 0.52, 0.69), HIV viral load (HR 1.20 per log_10_ increase; 95% CI: 1,01, 1.43) and cumulative exposure to lopinavir (HR 1.12 per additional year of use, 95% CI: 1.02, 1.22) were also associated with CKD in the multivariable model (Table 3). Liver fibrosis also demonstrated a trend towards greater risk of CKD (HR 1.50; 95% CI: 0.98, 2.30). In a secondary analysis, including hypertension and diabetes from the model did not appreciably change the results (HR 2.02; 95% CI: 1.36, 2.99), although tenofovir use was now associated with CKD (HR 1.12 per additional year of use, 95% CI: 1.00, 1.25). The effect of HCV co-infection did not differ between those who had IDU as a HIV risk factor and those who did not (*p*-value for interaction term, 0.94).

**Table 3:** Crude and adjusted Cox proportional hazards models for chronic kidney disease in the Canadian Observational Cohort Collaboration ^a^

	Unadjusted HR (95% CI)	Adjusted HR^c^ (95% CI)	Adjusted HR^d^ (95% CI)
Hepatitis C virus co-infection	2.49 (1.79, 3.48)	1.97 (1.33, 2.90)	2.02 (1.36, 2.99)
Female sex	1.56 (1.07, 2.28)	2.16 (1.42, 3.28)	2.12 (1.39, 3.23)
Age ≤ 40 years, per 5 year increase ^b^	0.97 (0.78, 1.21)	0.84 (0.67, 1.07)	0.82 (0.65, 1.04)
Age > 40 years, per 5 year increase ^b^	1.59 (1.45, 1.74)	1.51 (1.35, 1.67)	1.45 (1.31, 1.62)
African/Caribbean ethnicity	0.55 (0.32, 0.94)	0.79 (0.43, 1.44)	0.72 (0.39, 1.32)
Baseline eGFR ≤100 mL/min/1.73 m^2^, per 10 mL/min/1.73 m^2^ increase ^b^	0.56 (0.49, 0.65)	0.60 (0.52, 0.69)	0.61 (0.52, 0.70)
Baseline eGFR >100 mL/min/1.73 m^2^, per 10 mL/min/1.73 m^2^ increase ^b^	0.86 (0.70, 1.06)	1.02 (0.80, 1.30)	1.02 (0.80, 1.28)
CD4^+^ cell count, per 100 cells/μL increase	0.92 (0.86, 0.99)	0.98 (0.91, 1.06)	0.97 (0.90, 1.05)
HIV viral load, per log_10_ copies/mL increase	1.18 (1.01, 1.38)	1.20 (1.01, 1.43)	1.20 (1.01, 1.42)
Year of cART initiation, per calendar year increase	0.99 (0.94, 1.05)	1.07 (0.99, 1.15)	1.07 (0.99, 1.15)
Tenofovir use, per cumulative year of use	1.17 (1.07, 1.29)	1.11 (0.99, 1.24)	1.12 (1.00, 1.25)
Atazanavir use, per cumulative year of use	1.16 (1.06, 1.28)	1.09 (0.98, 1.21)	1.10 (0.99, 1.23)
Lopinavir use, per cumulative year of use	1.12 (1.03, 1.22)	1.12 (1.02, 1.22)	1.12 (1.02, 1.23)
Liver fibrosis (APRI ≥1.5)	1.59 (1.08, 2.34)	1.50 (0.98, 2.30)	1.50 (0.98, 2.30)
Hypertension	1.69 (0.96, 2.99)	N/A	1.70 (0.91, 3.17)
Diabetes	3.42 (2.29, 5.10)	N/A	1.47 (0.96, 2.26)

*APRI* aspartate aminotransferase to platelet ratio index, *cART* combination antiretroviral therapy, *CI* confidence interval, *eGFR* estimated glomerular filtration rate, *HR* hazard ratio, *N/A* not available

^a^ Multiple imputation used for missing data

^b^ Age and baseline eGFR were modeled with a linear spline

^c^ Hypertension and diabetes excluded from the model

^d^ Hypertension and diabetes included in the model

Results from all sensitivity analyses were quantitatively similar to the main findings. With an inverse-probability weighted sample to account for potential selection bias, the adjusted HR for HCV co-infection was similar (HR 2.01; 95% CI: 1.34, 3.01; see Additional file 1: Table S2). When using age as the time scale, rather than time from cART initiation, the adjusted HR increased slightly (HR 2.14; 95% CI: 1.44, 3.20; see Additional file 1: Table S3). When we considered person-time prior to a patient’s first positive HCV antibody or molecular test to be unexposed, the adjusted effect estimate was slightly attenuated (HR 1.82; 95% CI: 1.22, 2.73). Estimates from our additional MI scenarios, where we assumed missing data patterns for hypertension depended on unobserved data, were robust to our missing data assumptions (see Additional file 1: Table S4).

## Discussion

In this analysis of HIV-infected individuals initiating cART, we found that HCV co-infection was associated with a shorter time to incident CKD using a confirmed eGFR-based definition. This finding was independent of traditional CKD risk factors, which have become more prevalent in HIV-infected patients in the modern cART era [15]. Results were consistent for all sensitivity analyses using different estimation techniques. The adjusted effect size for HCV co-infection was relatively larger than other important CKD risk factors such as hypertension, diabetes, and exposure to nephrotoxic agents, such as tenofovir, atazanavir and lopinavir/ritonavir. These findings support the current guidelines for the diagnosis and management of CKD among HIV patients, which recommend annual monitoring of kidney function among those co-infected with HCV [37]. Identification of HIV patients at greatest risk of developing CKD will help targeted implementation of preventative and therapeutic strategies to slow renal function decline and determine which patients may benefit to switching newer cART regimens with safer renal profiles [38].

This is the first study to estimate the incidence of CKD among HIV-infected patients after initiating cART, which we estimated at 14 per 1000 PYFU. Previous studies have included a combination of treatment-naïve and experienced patients [39, 40], failed to use a confirmatory eGFR measurement [41, 42], or did not perform multivariable analyses [43]. Generally, results from our study were consistent with findings from other observational HIV cohorts. In a follow-up of HIV patients enrolled in the Strategies for Management of Antiretroviral Therapy trial, co-infected patients were 72% more likely to develop CKD and end-stage renal disease (ESRD), relative to HIV mono-infected patients [44]. This study also found that hepatitis B virus co-infection was also strongly associated with CKD and ESRD, which we did not observe in our analysis (data not shown). An analysis of data from EuroSIDA, which had a similar CKD incidence rate (15 per 1000 PYFU) as observed in CANOC, also demonstrated a two-fold increase in the risk of eGFR-based CKD for HCV co-infected patients, although there was no evidence of a dose-response relationship with increasing HCV viral load [40]. In the Women’s Interagency HIV Study, HCV co-infection was associated with a decline in eGFR among women with existing CKD, suggesting our findings are not restricted to males who make up the majority of participants in observational HIV cohorts [39].

In our analyses, we identified several additional CKD risk factors such female sex, increasing age, lower eGFR at treatment initiation, increasing HIV viral load, and cumulative exposure to tenofovir and lopinavir, which have previously been identified [45, 46]. Unlike other settings in North America, however, we did not find an association between African/Caribbean ethnicity and incident CKD [47, 48]. African/Caribbean patients in CANOC were less likely to be co-infected and started cART at a younger age (median 37 years vs. 40 years), compared to non-African/Caribbean individuals. It is likely that this reduced risk in CKD among African/Caribbean individuals observed in this study can be attributed to the use of cART which substantially reduces the risk of HIVAN, a common consequence of untreated HIV infection in this population [49].

There are several possible mechanisms by which HCV increases the risk of CKD. Declines in renal function could be brought about HCV-induced glomerular diseases, namely membranoproliferative glomerulonephritis, which is driven by deposition of mixed immune complexes in the glomerular units of the kidney [8]. Indeed, a biopsy study of 249 HIV patients with CKD found that co-infected patients were twice as likely to have histopathologic evidence of immune-complex glomerulonephritides, compared to HIV mono-infected patients [50]. Alternatively, HCV co-infection may be associated with CKD by increasing the prevalence of traditional CKD risk factors. In HIV-infected populations, co-infection has been associated with insulin resistance [51] and increases in carotid intima-media thickness and atherosclerosis [52], early markers for diabetes and cardiovascular disease, respectively. Lastly, as with HIV, there is evidence that persistent HCV replication can result in systematic inflammation and immune activation leading to endothelial dysfunction, which is prevalent in patients with severe renal failure [53, 54].

As with previous research, we were unable to account for the possible role that current IDU practices may have on CKD. Active IDU is prevalent among co-infected individuals and has been associated with increases in serum creatinine concentration [55]. Previous research in co-infected populations have demonstrated that frequent use of injection cocaine, a known nephrotoxic stimulant, may explain part of the association between chronic HCV and CKD observed in previous studies [56]. In our analysis, IDU as a HIV risk factor was strongly collinear with HCV co-infection and therefore not included in multivariable analyses. Furthermore, past IDU is often a poor proxy and imperfect measure for current IDU behaviour. In the Canadian Co-Infection Cohort, for example, 81% of participants have an IDU risk factor, but only 34% remained active users [25]. We found no evidence, however, that the role of HCV co-infection differed between those with and without a past IDU risk factor, as has been suggested for HIV treatment outcomes, such as virologic suppression, CD4^+^ recovery and all-cause mortality [31]. Co-infected patients may also experience higher levels of additional CKD risk factors, such as NSAID use, smoking, alcohol abuse, and food insecurity, compared to HIV mono-infected patients. These variables were not available in CANOC and thus we cannot ascertain how much they contribute to our observed association.

If HCV co-infection is an important driver of CKD in HIV-infected patients in the modern cART era, then CKD is an extrahepatic complication that can potentially be impacted with wider uptake of HCV treatment with new direct acting antivirals. Indeed, a recent study found that HCV-HIV co-infected responders who were successfully treated with pegylated-interferon and ribavirin had a 60% reduction in the risk of renal events, compared to individuals who failed treatment [57]. These findings provide further evidence of a direct effect of ongoing HCV viral replication on CKD etiology in HIV-infected populations.

Our study has several limitations. First, we excluded a large proportion of patients who did not have a baseline serum creatinine measurement. These patients, who were more likely to be female, white and be HCV co-infected, were excluded because we could not determine if they had prevalent CKD at cART initiation. In a sensitivity analyses where included patients were weighted by their inverse-probability of being selected into the analysis, we found little evidence of selection bias as a result of our exclusion criteria. Second, the use of a single HCV antibody serology, without RNA testing confirmation, as part of the HCV exposure definition may result in misclassification, as approximately 20% of individuals will spontaneously clear their HCV infection within a year of exposure and will no longer be viremic. This misclassification is likely non-differential with respect to time to incident CKD and biases our findings towards the null. Third, CANOC patients exposed to HCV may have subsequently initiated treatment and could have developed a sustained virologic response. We expect this misclassification of person-time to be small as both the uptake of HCV treatment and SVR rates among HIV co-infected patients in the pre direct-acting antiviral era are low [25]. Fourth, given the asymptomatic nature of chronic HCV infection, we assumed HCV infection was present at baseline and subjects did not seroconvert after starting cART even if their first positive test or diagnosis occurred after treatment initiation. This assumption may not be valid if subjects engaged in high-risk behavior after starting cART, however previous studies have shown that HCV infection is often acquired prior to starting treatment and seroconversion is relatively rare when on stable cART [25, 58]. Fifth, we had no information regarding quantitative HCV viral load to further evaluate the association between viral replication and CKD, as has been suggested earlier [44]. This has limited our ability to assess the plausibility of HCV as an etiologic CKD risk factor. Finally, we did not have data on proteinuria measurements preventing the use of more rigorous CKD staging definitions [59].

## Conclusions

The risk of CKD was more than two-fold greater in HCV co-infected compared to HIV mono-infected patients. Whether this risk is a solely a direct effect of HCV viral replication or is in part related to other associated risk factors, such as IDU, remains to be determined. Regardless, HCV-HIV co-infected individuals should be regularly screened for CKD to identify those who may require modifications to potentially nephrotoxic HIV treatments and those who may benefit from interventions to reduce further decline in kidney function such as HCV treatment with new direct-acting antiviral therapy.

## Additional file

Additional file 1: Supplemental content (**Tables S1 to S4**). (PDF 187 kb)

## Abbreviations

AIDS: Acquired immune deficiency syndrome
APRI: Aspartate aminotransferase to platelet ratio index
CANOC: Canadian Observational Cohort
cART: Combination antiretroviral therapy
CI: Confidence interval
CKD: Chronic kidney disease
eGFR: Estimated glomerular filtration rate
ESRD: End-stage renal disease
HCV: Hepatitis C virus
HIV: Human immunodeficiency virus
HIVAN: HIV-associated nephropathy
HR: Hazard ratio
IDU: Injection drug use
IQR: Interquartile range
MI: Multiple imputation
NSAID: Nonsteroidal anti-inflammatory drug
PH: Proportional hazards
PYFU: Person-years of follow-up
SCr: Serum creatinine
