# Supplementary material for: Hepatitis C co-infection is associated with an increased risk of incident chronic kidney disease in HIV-infected patients initiating combination antiretroviral therapy
Source: BMC Infect Dis. 2017 Apr 4;17:246. doi: 10.1186/s12879-017-2350-8 (PMC5381089; doi:10.1186/s12879-017-2350-8)
Supplement: Additional file 1: — Supplemental content (Tables S1 to S4). (PDF 187 kb) [file 12879_2017_2350_MOESM1_ESM.pdf]

Hepatitis C Co-Infection is Associated with an Increased Risk of Incident Chronic Kidney Disease in HIV-Infected Patients Initiating Combination Antiretroviral Therapy

Supplemental Digital Content

Authors:

Carmine Rossi[1], Janet Raboud[2,3], Sharon Walmsley[3], Curtis Cooper[4], Tony Antoniou[5], Mark Hull[6,7], Jason Chia[6], Robert S. Hogg[6,8], Erica E.M. Moodie[9] and Marina B. Klein[10], for the Canadian Observational Cohort (CANOC) Collaboration.

Affiliations:

[1] Research Institute of the McGill University Health Centre, Montréal, Canada

[2] Dalla Lana School of Public Health, University of Toronto, Toronto, Canada

[3] Toronto General Hospital Research Institute, University Health Network, Toronto, Canada

[4] The Ottawa Hospital, General Campus, Ottawa, Canada

[5] St. Michael's Hospital, University of Toronto, Toronto, Canada

[6] BC Centre for Excellence in HIV/AIDS, Vancouver, Canada

[7] Faculty of Medicine, University of British Columbia, Vancouver, Canada

[8] Faculty of Health Sciences, Simon Fraser University, Vancouver, Canada

[9] Department of Epidemiology, Biostatistics and Occupational Health, McGill University, Montréal, Canada

[10] Division of Infectious Diseases and Chronic Viral Illness Service, McGill University Health Centre, Montréal, Canada

Table S1: Comparison of baseline characteristics between included and excluded participants

	Included (n = 2595)	Excluded (n = 6385)
Median age (IQR), years	40 (33, 46)	40 (32, 47)
Male sex (%)	2195 (85%)	5114 (80%)
African/Caribbean (%) [a]	368 (24%)	421 (11%)
Median year of cART initiation (IQR)	2007 (2004, 2009)	2007 (2003, 2010)
Median HIV viral load (IQR), log_{10} copies/mL	4.9 (4.4, 5.2)	4.8 (4.3, 5.1)
Median CD4+ count (IQR), cells/µL	210 (100, 320)	240 (140, 370)
Hepatitis C virus co-infection (%) [b]	484 (20%)	1723 (29%)
IDU as HIV risk factor (%) [c]	389 (18%)	1632 (32%)
Tenofovir use (%)	1410 (54%)	4533 (71%)
Atazanavir use (%)	666 (26%)	1724 (27%)
Lopinavir use (%)	471 (18%)	830 (13%)
Hypertension (%) [d]	74 (12%)	113 (9%)
Diabetes (%) [e]	119 (5%)	260 (5%)
Cohort Province (%)		
British Columbia	1103 (43%)	3192 (50%)
Ontario	838 (32%)	2107 (33%)
Québec	654 (25%)	1086 (17%)

cART = combination antiretroviral therapy; HCV = hepatitis C virus; IDU = injection drug use; IQR=interquartile range.

[a] Due to missing data, denominator is 1550 among included participants and 3959 among excluded participants.

[b] Denominator is 2462 among included participants and 5929 among excluded participants.

[c] Denominator is 2164 among included participants and 5156 among excluded participants.

[d] Denominator is 622 among included participants and 1210 among excluded participants.

[e] Denominator is 2453 among included participants and 5425 among excluded participants

Table S2: Adjusted Cox proportional hazards models for chronic kidney disease in the Canadian Observational Cohort Collaboration using stabilized inverse probability of selection weights [a,b]

	Adjusted HR (95% CI)
Hepatitis C Virus Co-Infection	2.01 (1.34, 3.01)
Female sex	2.14 (1.39, 3.30)
Age ≤ 40 years, per five year increase [c]	0.80 (0.61, 1.05)
Age > 40 years, per five year increase [c]	1.43 (1.28, 1.62)
African/Caribbean ethnicity	0.78 (0.42, 1.45)
Baseline eGFR ≤ 100 mL/min/1.73 m^2, per 10 mL/min/1.73 m^2 increase [c]	0.63 (0.54, 0.73)
Baseline eGFR > 100 mL/min/1.73 m^2, per 10 mL/min/1.73 m^2 increase [c]	1.02 (0.81, 1.30)
$CD4^+$ cell count, per 100 cells/µL increase	0.99 (0.88, 1.11)
HIV viral load, per $\log_{10}$ copies/mL increase	1.23 (1.02, 1.47)
Year of cART initiation, per calendar year increase	1.07 (0.98, 1.15)
Tenofovir use, per cumulative year of use	1.12 (0.99, 1.27)
Atazanavir use, per cumulative year of use	1.11 (0.98, 1.26)
Lopinavir use, per cumulative year of use	1.12 (1.02, 1.22)
Liver fibrosis (APRI ≥ 1.5)	1.48 (0.99, 2.22)
Hypertension	1.78 (0.95, 3.36)
Diabetes	1.54 (0.97, 2.43)

APRI = aspartate aminotransferase to platelet ratio index; cART = combination antiretroviral therapy; CI = confidence interval; eGFR = estimated glomerular filtration rate; HR = hazard ratio.

[a] Multiple imputation used for missing data.

[b] Mean stabilized inverse-probability weight was 1.00, with a standard deviation of 0.25. The range of the weights was 0.48 to 1.80. Weights were stabilized using the probability of being selected into the study in the numerator.

[c] Age and baseline eGFR were modeled with a linear spline.

Table S3: Adjusted Cox proportional hazards models for chronic kidney disease in the Canadian Observational Cohort Collaboration using age as the time scale [a]

	Adjusted HR (95% CI)
Hepatitis C Virus Co-Infection	2.14 (1.44, 3.20)
Female sex	2.05 (1.34, 3.15)
African/Caribbean ethnicity	0.75 (0.40, 1.39)
Baseline eGFR ≤ 100 mL/min/1.73 m^2, per 10 mL/min/1.73 m^2 increase [b]	0.62 (0.54, 0.72)
Baseline eGFR > 100 mL/min/1.73 m^2, per 10 mL/min/1.73 m^2 increase [b]	0.97 (0.77,1.23)
$CD4^+$ cell count, per 100 cells/µL increase	0.96 (0.89, 1.03)
HIV viral load, per log_{10} copies/mL increase	1.19 (1.00, 1.42)
Year of cART initiation, per calendar year increase	1.12 (1.05, 1.19)
Tenofovir use, per cumulative year of use	1.03 (0.94, 1.13)
Atazanavir use, per cumulative year of use	1.06 (0.96, 1.17)
Lopinavir use, per cumulative year of use	1.06 (0.98, 1.14)
Liver fibrosis (APRI ≥ 1.5)	1.32 (0.87, 2.02)
Hypertension	1.72 (0.93, 3.16)
Diabetes	1.36 (0.87, 2.14)

APRI = aspartate aminotransferase to platelet ratio index; cART = combination antiretroviral therapy; CI = confidence interval; eGFR = estimated glomerular filtration rate; HR = hazard ratio.

[a] Multiple imputation used for missing data.

[b] Baseline eGFR was modeled with a linear spline.

Table S4: Sensitivity analysis of the hazard ratio estimating the association between hepatitis C co-infection and incident chronic kidney disease under several missing not at random (MNAR) scenarios for hypertension

Shift Parameter [a], δ	Adjusted HR [b] (95% CI)
δ = -3.0	2.12 (1.43, 3.14)
δ = -2.0	2.12 (1.43, 3.16)
δ = -1.5	2.12 (1.42, 3.15)
δ = -1.0	2.11 (1.41, 3.16)
δ = -0.5	2.09 (1.40, 3.13)
δ = 0 (Initial multiple imputation analysis)	2.02 (1.36, 2.99)
δ = 0.5	2.03 (1.36, 3.04)
δ = 1.0	2.01 (1.35, 2.99)
δ = 1.5	2.00 (1.34, 2.96)
δ = 2.0	1.99 (1.34, 2.95)
δ = 3.0	1.98 (1.33, 2.93)

CI = confidence interval; HR = hazard ratio.

[a] The shift parameter, δ, is an increase in the log odds of the probability of being normotensive for those with missing hypertension data, given observable covariates in the imputation model. δ = 0, therefore, refers to an analysis where the data is assumed to be missing at random (MAR), conditional on observable covariates. For $\delta < 0$, we assumed that those with missing data on hypertension were more likely to be hypertensive. For $\delta > 0$, we assumed that those with missing data on hypertension were more likely to be normotensive.

[b] For each sensitivity analysis, *m*=10 data sets were imputed using fully conditional specification, as detailed in the methodology section, and regression results were pooled using Rubin's rules.
